# Impact of pre-transplant dialysis modality on the outcome and health-related quality of life of patients after simultaneous pancreas-kidney transplantation

**DOI:** 10.1186/s12955-020-01545-3

**Published:** 2020-09-10

**Authors:** Uwe Scheuermann, Sebastian Rademacher, Nora Jahn, Elisabeth Sucher, Daniel Seehofer, Robert Sucher, Hans-Michael Hau

**Affiliations:** 1 Department of Visceral, Transplantation, Thoracic and Vascular Surgery, University Hospital of Leipzig, Leipzig, Germany; 2 Department of Anesthesiology and Intensive Care Medicine, University Hospital of Leipzig, Leipzig, Germany; 3 Department of Gastroenterology, University Hospital of Leipzig, Leipzig, Germany; 4 Department of Visceral, Thoracic and Vascular Surgery, University Hospital and Faculty of Medicine Carl Gustav Carus, Technische Universität Dresden, Dresden, Germany

**Keywords:** Health-related quality of life, Hemodialysis, Outcome, Peritoneal dialysis, Simultaneous pancreas-kidney transplantation, Survival

## Abstract

**Background:**

Simultaneous pancreas-kidney transplantation (SPKT) profoundly improves the health-related quality of life (HRQoL) of recipients. However, the influence of the pre-transplant dialysis modality on the success of the SPKT and post-transplant HRQoL remains unknown.

**Methods:**

We analyzed the surgical outcome, long-term survival, as well as HRQoL of 83 SPKTs that were performed in our hospital between 2000 and 2016. Prior to transplant, 64 patients received hemodialysis (HD) and nineteen patients received peritoneal dialysis (PD). Physical and mental quality of life results from eight basic scales and the physical and mental component summaries (PCS and MCS) were measured using the Short Form 36 (SF-36) survey.

**Results:**

Peri- and postoperative complications, as well as patient and graft survival were similar between the two groups. Both groups showed an improvement of HRQoL in all SF-36 domains after transplantation. Compared with patients who received HD before transplantation, PD patients showed significantly better results in four of the eight SF-36 domains: physical functioning (mean difference HD - PD: − 12.4 ± 4.9, P = < 0.01), bodily pain (− 14.2 ± 6.3, *P* < 0.01), general health (− 6.3 ± 2.8, *P* = 0.04), vitality (− 6.8 ± 2.6, P = 0.04), and PCS (− 5.2 ± 1.5, *P* < 0.01) after SPKT. In the overall study population, graft loss was associated with significant worsening of the HRQoL in all physical components (each P < 0.01).

**Conclusions:**

The results of this analysis show that pre-transplant dialysis modality has no influence on the outcome and survival rate after SPKT. Regarding HRQoL, patients receiving PD prior to SPKT seem to have a slight advantage compared with patients with HD before transplantation.

## Introduction

Simultaneous pancreas-kidney transplantation (SPKT) offers considerable survival benefits for patients with insulin-dependent diabetes mellitus and end-stage renal disease (ESRD), because it restores long-term glycemic control and can reduce secondary diabetic complications [1–6].

Due to organ shortage, most of these patients must undergo long-term renal replacement therapy (RRT) prior to SPKT. With hemodialysis (HD) and peritoneal dialysis (PD), two efficient RRTs are available pre-transplant. However, both dialysis modalities substantially influence patients’ health, as well as their ability to work and participate in social activities. Conventional HD is usually performed intermittently three times a week in a dialysis center. During HD, uremic toxins are removed from the blood extracorporeally through a filter, which is generally considered as a stressful procedure, with an increased risk of cardiovascular diseases [7, 8]. By contrast, PD uses the peritoneum as a filter through which substances are exchanged with the blood. In general, PD offers greater flexibility as it can be performed at different time points, almost always at home and independent of medical staff, although it carries the risk of metabolic complications related to systemic glucose absorption from the dialysate (e.g. hyperlipidemia and decompensation of diabetes mellitus), and catheter-associated complications. PD catheter infection can lead to subsequent peritonitis, which remains the major cause of morbidity and mortality in PD patients [7, 9].

Apart from mere graft and patient survival rates after transplantation, measuring the health-Related Quality of Life (HRQoL) has become an issue of further interest as it provides an overall view of the impact of the disease process on psychosocial status. Monitoring the HRQoL helps to define how disease symptoms affect the patient’s life, physical and mental functioning, and their ability to cope with the new life situation after transplantation.

Recent studies have shown that patients generally experience better quality of life after SPKT compared with dialysis patients [4, 10–13]. However, the influence of dialysis modality prior to SPKT on the surgical outcome and HRQoL after transplantation is less clear. Thus, the current study has sought to analyze the outcome and HRQoL in diabetic patients with ESRD undergoing SPKT regarding pre-transplant dialysis modality.

## Methods

### Study population

Medical data from patients with insulin-dependent diabetes mellitus (type 1 or 2) and ESRD who received SPKT at the University Hospital of Leipzig between 2000 and 2016 were retrospectively analyzed. Our data source comprised a prospectively collected electronic database. Approval for this analysis was granted by the local ethics committee [AZ: Nr: 111–16-14,032,016]. Patients undergoing pre-emptive transplantation, re-transplants, living donor kidney transplantation, those aged younger than 18 years, and those with missing data were excluded from the study.

### Outcome measures

Special emphasis was placed on the outcome of dialysis modality before transplantation (HD versus PD), recipient and donor characteristics, intra- and postoperative variables and complications such as patient, graft as well as HRQoL outcomes before (during dialysis) and after transplantation. Characteristics included age, gender, body mass index (BMI, weight in kg/height in m^2^), duration of insulin-dependent diabetes mellitus, duration of dialysis, and time on the waiting list. Cardiovascular disease included information about peripheral arterial obstructive disease and coronary heart disease (coronary artery bypass graft or stent). Peri- and post-transplant data included information on cold ischemia time of the kidney/pancreas, immunosuppressive therapy as well as patient and organ graft function.

Furthermore, complications occurring during the first 3 months after transplantation were analyzed. Surgical complications were defined as the need for relaparotomy within the first 3 months after transplantation. Intra-abdominal infections were defined as the development of infected fluid collection that required an intervention and/or antibiotic drug therapy. Gastrointestinal bleedings were defined as bleedings requiring relaparotomy or endoscopy, or patients suffering from sudden anemia combined with either melena or hematemesis. Other bleedings comprising intra-abdominal hemorrhages, were diagnosed by CT scan or relaparotomy performed due to acute anemia.

### Health-related quality of life (HRQoL)

For measuring the HRQoL in this study, the most commonly-used and internationally-validated Medical Outcomes Study 36-Item Short Form Health Survey (SF-36) questionnaire was used. Patients were asked to evaluate their HRQoL before transplantation (undergoing dialysis), such as 1 year after transplantation. Therefore, SF-36 forms were completed at different time points after transplantation, but not earlier than 1 year after transplantation.

HRQoL was evaluated in a second assessment through written HRQoL questionnaires (SF-36) sent to all patients with an invitation to complete a quality-of-life survey retrospectively. The SF-36 survey was sent by mail to patients’ home addresses. Additionally, patients were interviewed via telephone or during a clinical visit, as indicated. Return envelopes were included free of charge. A period of 2 weeks was envisaged for returning questionnaires. All patients who had not returned the questionnaire after 2 weeks were contacted a second time via mail or telephone within another 14 days. Altogether, patients had 4 weeks to submit their responses. Prior to the study, informed consent of all patients and the local ethics committee [AZ: Nr: 111–16-14,032,016] was obtained. Because the HRQoL assessment was performed separately, it was not possible to correlate HRQoL scores with all clinical data. The average time span between transplantation and completion of the questionnaires was 8.6 ± 2.9 years.

The SF-36 has 36 questions (without specific one for renal failure or diabetes) that assess the ability to perform vigorous activities and activities for daily living and participate in social, family and occupational activities. Eight scales/dimensions describe domains of physical functions (PF, ten items), role limitations due to physical problems (PR, four items), bodily pain (BP, two items), a general perception of health (GH, five items), energy and vitality (VIT, four items), social functions (SF, two items), role limitations due to emotional problems (RE, three items), and mental health (MH, five items). The subscales can be combined into two summation scales measuring the overall physical and mental HRQoL: a physical component summary (PCS = PF + PR + BP + GH) and a mental component summary (MCS = VIT + SF + RE + MH). Subscale scores were transformed to a 0–100 scale, with 0 representing the least to 100 representing the greatest well-being.

### Surgical techniques

The pancreas and kidney grafts were procured according to the guidelines provided by Eurotransplant (ET) [14]. As described earlier, the pancreas was explanted in a no-touch technique [15]. An illiac y-graft was used for reconstruction of the superior mesenteric and the lienal artery. The pancreas was transplanted transabdominally using a standard technique with an intraperitoneal location in the right iliac fossa [16]. Exocrine drainage was carried out with a side-to-side duodenojejunostomy [3, 17]. Kidneys were transplanted transabdominally into the left iliacal fossa. The ureter was implanted into the bladder according to the Lich-Gregoir technique using a double J intra-ureteral splint [16].

### Immunosuppression

Immunosuppressive therapy comprised an induction therapy with the interleukin-2 receptor antagonist basiliximab or antithymocyte globulin, followed by a triple maintenance immunosuppression consisting of calcineurin inhibitors (tacrolimus or cyclosporine), and/or antimetabolites (mycofenolate mofetil or sirolimus) and tapered steroids (prednisolone).

### Statistical analysis

Baseline data are presented as mean values with the standard deviation (SD), minimum or maximum range such as the proportion percentage (%). Baseline data were compared with appropriate statistical significance test including the Student’s t–test, χ2, analysis of variance (ANOVA), Kruskal-Wallis and Wilcoxon–Mann–Whitney tests. Analysis of covariance (ANCOVA) was used to adjust the difference between the two groups after SPKT, with hemodialysis as one predictor and baseline measurements of HRQoL domains (before SPKT) as the other. Survival rates were calculated using the Kaplan-Meier analysis and the log-rank test was applied to test statistical significance. Graft survival was calculated as the time from initial transplant to graft failure (re-start of dialysis), censoring for death with a functioning graft and grafts still functioning at time of analysis. Patient survival is defined as time from transplant to patient death, censoring for patients still alive at time of analysis. If a recipient was alive or lost to follow-up at time of last contact, then survival time was censored at time of last contact. Multivariate Cox proportional hazards analysis was applied to assess independent predictors of patient death and pancreas graft failure, including clinically-relevant variables and/or those presenting *P* ≤ 0.15 in univariate analysis: recipient gender, BMI and age, dialysis modality (HD versus PD), time on dialysis, years of diabetes mellitus, concomitant cardiovascular disease and surgical complications. SPSS software (SPSS Inc., Chicago, Illinois, USA, version 21.0) was used for statistical analysis and graphs. A *P* value < 0.05 was considered as statistically significant.

## Results

### Baseline characteristics

The overall study population included 83 patients receiving SPKT in our department between 2000 and 2016. At the time of transplantation, 64 patients obtained HD and nineteen patients used PD. Continuous ambulatory peritoneal dialysis was used by fourteen patients (74%) and automatic peritoneal dialysis was used by five patients (26%) among the PD group. Recipient, donor and graft characteristics according to the different dialysis types prior to transplantation are summarized in Table 1**.** The two groups were similar for the majority of their pre-transplant characteristics, while the number of female recipients was higher in the PD group (*P* = 0.017), and a history of depression was more frequent in the HD group (*P* = 0.049). HLA mismatches, post-transplant immunosuppressive regimes and lengths of hospital stay did not show significant differences between the two groups (data not shown).

**Table 1:** Demographic and clinicopathologic characteristics of the study cohort prior to transplantation. Data are shown as mean ± SD. ALT, antilymphocyte globulin; ATG, anti-thymocyte globulin; BMI, body mass index; CMV, cytomegalovirus; D+, donor positive; R+ recipient positive; HbA1c, glycated hemoglobin; HD, hemodialysis; IL-2 RA, Interleukin-2 receptor antagonist; PD, peritoneal dialysis; SPKT, simultaneous pancreas-kidney transplantation

Variables	HD (*N* = 64)	PD (*N* = 19)	*P*-value
Recipient			
Age, years	43.8 ± 9.1	43.2 ± 9.7	0.845
Gender			
Male	40 (62. 5%)	6 (31.6%)	0.017
Female	24 (37.5%)	13 (68.4%)	
BMI, kg/m^2^	25.8 ± 4.4	24 ± 3.6	0.105
Duration diabetes mellitus, years	27.1 ± 8.4	26.1 ± 8.6	0.685
Time on dialysis, months	30.5 ± 21.3	27.0 ± 22.1	0.536
HbA1c pre-transplantation, %	7.7 ± 1.8	7.7 ± 1.2	0.940
Time on waiting list, months	10.9 ± 13.6	7.2 ± 6.9	0.242
Comorbidities			
Diabetic retinopathy	56 (87.5%)	13 (68.4%)	0.050
Diabetic neuropathy	39 (60.9%)	11 (57.9%)	0.812
Arterial obstructive disease	12 (18.8%)	2 (10.5%)	0.401
Coronary heart disease	21 (32.8%)	2 (10.5%)	0.050
Depression	21 (32.8%)	2 (10.5%)	0.049
Taking aspirin pre-SPKT	23 (35.9%)	9 (47.4%)	0.367
Donor			
Age, years	24.1 ± 11.8	19.2 ± 7.8	0.109
Gender			
Male	37 (57.8%)	14 (73.3%)	0.212
Female	27 (42.2%)	5 (26.3%)	
BMI, kg/m^2^	22.4 ± 3.1	22.1 ± 2.8	0.987
Graft			
Kidney cold ischemia, hours	11.0 ± 3.3	11.1 ± 2.6	0.978
Pancreas cold ischemia, hours	10.1 ± 1.9	10.9 ± 3.9	0.294
CMV status			
CMV D +	31 (48.4%)	11 (64.7%)	0.211
CMV R +	36 (56.3%)	13 (68.4%)	0.343
Induction Therapy			
ALG/ ATG	54 (84.4%)	15 (78.9%)	0.864
IL2-RA	7 (10.9%)	3 (15.8%)	0.568
None	3 (4.7%)	1 (5.3%)	0.916

### Outcome

#### Complications

There were no significant differences in the frequency of peri- and postoperative complications between HD and PD SPKT recipients **(**Table 2**).** The global relaparotomy rate was similar between the two groups (HD: 35% versus PD: 36%; *P* = 0.77).

**Table 2:** Post-transplant complications and causes of relaparotomy. CMV, cytomegalovirus; GI, gastrointestinal; HD, hemodialysis; PD, peritoneal dialysis; SPKT, simultaneous pancreas-kidney transplantation

Variables	HD (N = 64)	PD (N = 19)	*P*-value
Acute combined graft rejection	12 (19%)	3 (16%)	0.769
Delayed graft function kidney	10 (16%)	2 (11%)	0.80
Anastomotic leak	2 (3%)	1 (5%)	0.66
Graft thrombosis	7 (11%)	4 (21%)	0.254
GI-bleeding	7 (11%)	1 (5%)	0.426
Other major bleeding	9 (14%)	2 (11%)	0.69
Intra-abdominal infection	13 (20%)	5 (26%)	0.577
Graft pancreatitis	11 (17%)	3 (16%)	0.88
CMV infection	21 (33.3%)	5 (26.3%)	0.564
Wound infections	9 (14%)	3 (16%)	0.851
Re-operation/ relaparotomy	23 (35%)	7 (36%)	0.77
Causes relaparotomy			
Infection	8 (13%)	2 (11%)	0.76
Bleeding	6 (9%)	1 (5%)	0.51
Thrombosis	7 (11%)	3 (16%)	0.54
Others	2 (3%)	1 (5%)	0.66

After transplantation, eighteen patients developed intra-abdominal infections (HD: 20% versus PD: 26%; *P* = 0.577). There were eight bacterial infections, two fungal infections and eight cultured positive for both bacteria and fungi. Intra-abdominal infections were complicated with graft pancreatitis in nine cases (HD: 10% versus PD: 11%; *P* = 0.89) and relaparotomy was necessary in ten cases (HD: 13% versus PD: 11%; *P* = 0.76). Four out of nineteen PD patients had a history of peritonitis during their time on dialysis, including two patients with two or more episodes of peritonitis. A history of peritonitis was not associated with an increased risk of complications after transplantation, whereby only one of these patients had an intra-abdominal infection and one patient developed pancreatitis post-transplant.

#### Patient and graft survival

**Fig. 1:**
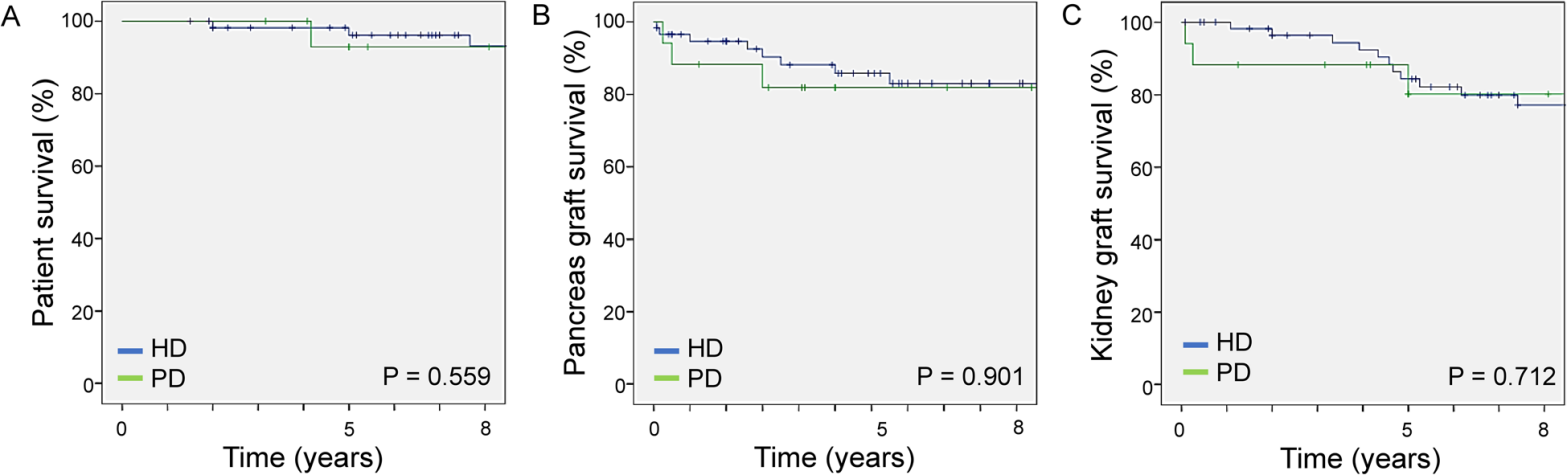
(**a**) Patient, (**b**) pancreas graft and (**c**) kidney graft survival after SPKT according to dialysis modality. HD, hemodialysis; PD, peritoneal dialysis

The 3- and 5-year patient survival rates for patients after SPKT showed no significant differences between the HD and PD group (98.2 and 96.1% in HD versus 92.9 and 92.9% in PD, respectively; *P* = 0.559). Similarly, the 1-, 3- and 5- year pancreas graft survival rates (94.6, 90.3 and 85.8% in HD versus 88.2, 81.9 and 81.9% in PD, respectively; *P* = 0.901) and kidney graft survival rates (98.2, 96.4 and 84.3% in HD versus 88.2, 88.2 and 80.2% in PD, respectively; *P* = 0.712) did not show any significant differences between the two groups **(**Fig. 1**)**.

As shown in Table 3, no significant differences were found between the type of pre-transplant dialysis modality and causes for patient death and graft failure. However, we noted a tendency of higher graft losses due to thrombosis in PD patients (HD: 4.7% versus PD: 15.8%; *P* = 0.10).

**Table 3:** Causes of patient death and pancreas and kidney graft loss after simultaneous pancreas-kidney transplantation

Variables	HD (N = 64)	PD (N = 19)	*P*-value
Patient death			
Total	12 (19%)	3 (16%)	0.78
Cardiovascular	6 (9.4%)	2 (10.5%)	0.88
Infection	4 (6.3%)	1 (5.3%)	0.87
Other	2 (3.1%)	0 (0%)	0.43
Pancreas graft failure			
Total	15 (23%)	5 (26%)	0.79
Rejection	3 (4.7%)	0 (0%)	0.33
Thrombosis	3 (4.7%)	3 (15.8%)	0.10
Bleeding	2 (3.1%)	0 (0%)	0.43
Infection	5 (7.8%)	2 (10.5%)	0.70
Other	2 (3.1%)	0 (0%)	0.43
Kidney graft failure			
Total	13 (20%)	3 (16%)	0.19
Rejection	6 (9%)	1 (5%)	0.32
Thrombosis	2 (3%)	1 (5%)	0.66
Infection	3 (5%)	1 (5%)	0.91
Other	2 (3%)	0 (0%)	0.45

Multivariate Cox regression analysis of the total study population revealed that the presence of cardiovascular disease is an independent predictor of patient death. Moreover, the preoperative presence of cardiovascular disease, recipient age, BMI, duration of pre-transplant dialysis and surgical complications could be identified as independent predictors of pancreas and kidney graft loss **(**Table 4**)**.

**Table 4:** Multivariate Cox regression analysis of predictors of patient death and pancreas graft loss. BMI, body mass index; HD, hemodialysis; HR (95CI), hazard ratio (95% confidence interval); PD, peritoneal dialysis

Variables	HR (95 CI)	*P*-value
Patient death		
Cardiovascular disease	6.12 (1.7–21.19	0.005
Dialysis modality (PD vs HD)	1.72 (0.3 7–7.71)	0.70
Recipient age	0.96 (0.90–1.04)	0.33
Recipient gender (male vs female)	1.36 (0.41–4.44)	0.60
Months on dialysis	1.01 (0.9–1.0)	0.25
Years of diabetes	1.01 (0.9–1.2)	0.65
Recipient BMI	1.02 (0.88–1.18)	0.75
Pancreas graft failure		
Cardiovascular disease	3.36 (1.14–9.89)	0.02
Dialysis modality (PD vs HD)	1.35 (0.43–4.27)	0.60
Recipient age	1.2 (0.99–1.12)	0.01
Recipient gender (male vs female)	2.01 (0.81–4.92)	0.129
Months on dialysis	0.98 (0.96–1.10)	0.001
Years of diabetes	1.02 (0.94–1.06)	0.95
Surgical complications	6.48 (2.66–15.74)	0.001
Recipient BMI	1.2 (1.08–1.35)	0.01
Kidney graft failure		
Cardiovascular disease	2.53 (0.82–7.46)	0.019
Dialysis modality (PD vs HD)	1.01 (0.28–3.59)	0.986
Recipient age	1.2 (0.99–1.12)	0.01
Recipient gender (male vs female)	2.1 (0.75–6.1)	0.15
Months on dialysis	0.96 (0.93–1.2)	0.001
Years of diabetes	1.1 (0.95–1.08)	0.74
Surgical complications	3.4 (1.5–6.9)	0.03
Recipient BMI	1.19 (1.06–1.33)	0.003

#### Health-related quality of life (HRQoL)

##### Impact of pre-transplant dialysis modality

Tables 5 compares the HRQoL scores of SPKT recipients according to their dialysis modality before and after transplantation. Both groups showed an improvement of HRQoL in all SF-36 domains after transplantation. There were statistically significant improvements in six of the eight SF-36 domains in the HD group, and in seven of the eight SF-36 domains in the PD group.

**Table 5:** Comparison of HRQoL between HD and PD patients before and after SPKT. Data are shown as mean ± SD. HD, hemodialysis; MCS, mental component summary; MD, mean difference; PCS, physical component summary; PD, Peritoneal dialysis; SF-36, Short Form 36; SPKT, simultaneous pancreas-kidney transplantation

	Groups						HD - PD			
SF-36 dimensions	HD (N = 64)			PD (N = 19)			Before SPKT		After SPKT	
	Before SPKT	After SPKT	*P*-value	Before SPKT	After SPKT	*P*-value	MD	*P*-value	MD	*P*-value
Physcial functioning	48.3 ± 13.3	68.9 ± 8.9	< 0.01	63.8 ± 17.7	81.3 ± 15.9	0.030	− 15.2 ± 7.1	0.028	− 12.4 ± 4.9	< 0.01
Role limitations- physical	38.3 ± 31.3	60.7 ± 25.4	0.040	56.2 ± 43.8	72.9 ± 19.8	< 0.01	− 17.9 ± 16.4	0.267	− 12.2 ± 9.1	0.49
Bodily Pain	44.2 ± 21.2	71.1 ± 8.5	< 0.01	64.1 ± 17.6	85.3 ± 9.8	< 0.01	− 19.9 ± 9.3	0.034	− 14.2 ± 6.3	< 0.01
General health	36.5 ± 16.1	61.6 ± 7.5	< 0.01	48.8 ± 7.0	67.9 ± 6.8	< 0.01	− 12.3 ± 6.3	0.053	− 6.3 ± 2.8	0.04
Social function	55.8 ± 17.6	65.2 ± 12.2	0.110	68.8 ± 11.6	73.9 ± 11.2	0.329	− 13.0 ± 7.4	0.077	− 8.7 ± 4.6	0.15
Vitality	42.7 ± 11.2	61.1 ± 7.4	< 0.01	48.8 ± 18.5	67.9 ± 5.4	< 0.01	− 6.1 ± 6.6	0.333	− 6.8 ± 2.6	0.04
Role limitations- emotional	44.4 ± 29.9	61.9 ± 28.8	0.122	66.7 ± 30.9	83.3 ± 22.5	0.178	− 22.3 ± 12.9	0.109	− 21.4 ± 10.3	0.36
Mental Health	51.4 ± 16.4	63.4 ± 5.8	0.010	49.5 ± 2.8	62.3 ± 8.2	< 0.01	− 1.9 ± 4.9	0.681	− 1.1 ± 2.7	0.96
PCS	34.9 ± 5.8	45.6 ± 4.6	< 0.01	43.1 ± 8.6	50.8 ± 2.5	< 0.01	− 8.2 ± 3.1	0.013	− 5.2 ± 1.5	< 0.01
MCS	40.2 ± 6.6	44.7 ± 4.5	0.042	41.6 ± 5.4	46.8 ± 3.8	0.021	−1.4 ± 2.9	0.624	− 2.1 ± 1.6	0.32

Before transplantation, benefits were seen for the PD patients, with statistically significant differences in terms of physical functioning (*P* = 0.028), bodily pain (*P* = 0.034), and in the physical component summary (PCS) (*P* = 0.013) compared with patients receiving HD prior to transplantation (Table 5). After SPKT, PD patients showed significantly better results in four of the eight SF-36 domains compared with HD patients: physical functioning (*P* < 0.01), bodily pain (P < 0.01), general health (*P* = 0.04), vitality (P = 0.04) and PCS (P < 0.01).

##### Impact of demographical and clinical variables

We separately analyzed the possible impact of three factors – age, gender and graft loss – on the HRQoL of the overall study population after SPKT **(**Table 6**)**. Regarding patients’ age, there was a significant difference in physical functioning (P = 0.04), general health (*P* < 0.01) and role limitations (*P* = 0.03). In the univariate analysis, patients’ gender showed no influence on HRQoL after SPKT. Graft loss (kidney or pancreas) led to a significant decrease in all physical components (each P < 0.01) and mental health status (*P* = 0.01) of the SF-36.

**Table 6:** Univariate analysis: impact of clinical and demographical variables on SF-36 results of all pati*e*nts after transplantation (*n* = 83). Data are shown as mean ± SD

SF-36 dimensions	Age			Gender			Graft loss		
	< 45 years	> 45 years	*P*-value	Male	Female	*P*-value	No	Yes	*P*-value
Physcial functioning	80.1 ± 11.2	69.8 ± 14.3	0.04	77.8 ± 13.4	74.2 ± 14.3	0.499	86.8 ± 6.8	65.6 ± 10.3	< 0.01
Role limitations- physical	73.5 ± 20.6	61.1 ± 13.1	0.09	69.3 ± 17.6	67.6 ± 20.1	0.308	76.3 ± 15.1	45.8 ± 10.2	< 0.01
Bodily Pain	80.1 ± 11.7	72.2. ± 9.6	0.1	79.8 ± 12.3	76.1 ± 11.1	0.416	81.8 ± 8.9	67.9 ± 7.7	< 0.01
General health	67.9 ± 5.8	59.8 ± 7.6	< 0.01	63.1 ± 9.1	66.4 ± 5.2	0.276	82.8 ± 8.5	46.7 ± 5.8	< 0.01
Social function	72.1 ± 10.4	63.9 ± 14.6	0.11	68.2 ± 15.6	70.0 ± 10.4	0.719	73.9 ± 11.2	65.0 ± 12.1	0.08
Vitality	66.2 ± 5.7	61.8 ± 9.2	0.15	67.3 ± 7.2	61.3 ± 6.7	0.047	66.4 ± 5.9	55.2 ± 5.1	0.06
Role limitations- emotional	82.1 ± 22.4	61.5 ± 23.7	0.03	68.9 ± 29.4	75.8 ± 21.6	0.545	80.1 ± 5.1	61.1 ± 20.3	0.09
Mental Health	63.3 ± 7.9	62.4 ± 5.6	0.66	63.7 ± 7.5	61.8 ± 6.3	0.498	65.1 ± 5.6	57.1 ± 1.3	0.01

## Discussion

### Post-transplant outcome and survival

Infectious complications remain a significant cause of morbidity and mortality after SPKT [3, 4, 6, 18]. A much-debated question is whether pre-transplant PD increases the risk for intra-abdominal infections after transplantation due to the risk of catheter-associated peritonitis. Binaut et al. showed a higher rate of parietal sepsis in PD patients after kidney transplantation alone (KTA) compared with patients who received HD prior to transplantation [19]. Martins et al. reported a significantly higher rate of pancreas loss due to infection among PD patients after SPKT (HD: 3.4% versus PD: 12.8%; *P* = 0.042), whereas ten out of 39 (25.6%) PD patients had peritonitis in their past [20]. By contrast, other studies have reported no difference in surgical outcome between HD and PD patients after SPKT [21–24]. Our analysis could not reveal significant differences in intra-abdominal infection rates after SPKT between the two groups, and there was no correlation between the incidence of pre-transplant peritonitis and post-transplant intra-abdominal infection. Besides dialysis modality, the incidence of intra-abdominal infection after SPKT may be related to other factors, namely surgical technique (especially pancreas-anastomosis), the length of hospital stays and the intensity of immunosuppression.

In the literature, there are reports of an increased graft loss in PD patients compared with HD patients post-transplant. After KTA, an increased graft loss in PD patients is reported, mainly due to renal vascular thrombosis [25, 26]. In a study investigating outcomes after SPKT, Martins et al. reported a significantly higher rate of thrombosis-driven relaparotomy in PD patients compared with HD patients. Moreover, they recognized a near significant higher rate of renal graft loss due to thrombosis in PD patients compared with HD patients [20]. In our study, no correlations were found between pre-transplant dialysis modality and patient or graft survival. Only the rate of pancreas graft failure due to thrombosis was non-significantly higher in the PD group (HD: 4.7% versus PD: 15.8%, *P* = 0.10). However, these data must be interpreted with caution, because other variables such as patient comorbidities (cardiovascular diseases, BMI), along with recipient age and time on dialysis might be additional factors that influence the outcome and survival.

### Health-related quality of life (HRQoL)

#### Pre-transplant HRQoL

Most published studies evaluating dialysis patients waiting for KTA show inconclusive results related to pre-transplant HRQoL and dialysis modality [27]. Previous publications have used a broad range of measures to assess HRQoL and analyzed patients with different demographic characteristics and medical history. Our findings indicate that PD is associated with a significantly better physical functioning and physical component summary before SPKT compared with patients receiving HD as a pre-transplant dialysis modality. Moreover, besides mental health, all other components of the SF-36 score were (although not significantly) higher in PD patients. The better reported quality of life among PD patients may stem from the positive effect of medical self-management through PD, which can be performed independently or with the help of a caregiver and develops a sense of personal control, which positively correlates with features of HRQoL. By contrast, conventional HD is a stressful process and time spent on a dialysis ward can contribute to a lower quality of life, as the number of hours in HD hinders professional and social functioning.

#### Post-transplant HRQoL

The HRQoL of patients improved in both study groups after transplantation, which underlines the therapeutic success of SPKT, besides medical outcomes and objective clinical parameters. This is in line with most published studies showing a significant improvement in patient-reported general HRQoL after SPKT and better diabetes-specific quality of life [4, 10–13].

Psychiatric disorders could influence the poor evaluation of the quality of life among HD patients [28]. HD carries the risk of vascular dehydration and hypotension following cerebellar hypoperfusion and may lead to cognitive impairment and cardiovascular diseases [29–31]. Sapilak revealed a strong negative correlation of HRQoL of HD patients with depression symptoms and anxiety [32]. In a study by Smith et al. evaluating 37 patients pre-transplant and 4 months after SPKT, approximately half of the patients showed an improvement of HRQoL, although one-third even experienced reduced HRQoL. The authors showed that pre-existing psychiatric disorders at the time of transplant were the only significant predictor of reduced quality of life following pancreas transplantation [33]. In line with these reports, in our study the rate of depression in patient history was significantly higher in the HD group (*P* = 0.049). However, with a small sample size and missing details about psychosocial status and longitudinal periodic assessment of depression disease, caution must be applied as the findings might not be representative.

In the literature, the relative importance of patients’ gender on HRQoL after transplantation is debated. In a study by Rajkumar et al., 12 months after transplantation (KTA or SPKT) most HRQoL scores (seven out of eight) were better for women than for men. Moreover, HRQoL scores of female recipients were not statistically different from the control group in the general population [12]. The authors postulated that differences in expectations and appreciation of improvements in health after transplantation, differences in social network and emotional support may provide an explanation for these findings. Other reports have favored men and shown that female gender is a negative predictive factor of HRQoL after SPKT [10, 33]. In a study by Martin et al. evaluating HRQoL in 60 male and 66 female SPKT patients, no lower scores for female patients were observed [11]. Different results in the literature may be explained by different follow-up periods, study populations and study designs/questionnaires. In our analysis, the number of female recipients was significantly higher in the PD group (*P* = 0.017), although we did not observe gender-based differences in the overall study population.

In the present study, we have shown that graft loss has a significant impact on HRQoL after SPKT due to a loss of independence from insulin therapy and freedom from dialysis. This is in line with previous publications in which graft loss has been associated with significant worsening of the HRQoL [4, 34, 35], and maintenance of two functioning grafts seems to be an independent predictor of higher post-transplant quality-of-life scores [11]. Furthermore, the results of Sutherland et al. indicate that achieving insulin independence improves QOL more than becoming dialysis-free [4].

### Limitations

There are some notable limitations of this study that should be mentioned, related to the data source and study design. The main limitations of the study are its retrospective nature, the heterogenous group size and the fact that all data collection was undertaken at one time, which make it difficult to evaluate the causality of the findings and associations. As such, there may be some amount of variation from year to year with changes in the outcome and level of HRQoL. Second, not only disease or dialysis modality determine one’s view of life quality, but also many non-disease factors (work, education, and other sociodemographic factors) play important additive role in the perception of HRQoL. The option for PD or HD normally depends on the patient’s condition (comorbid situations, vascular and peritoneal conditions), autonomy and convenience, as well as the health care system (dialysis center factors).

## Conclusions

Both dialysis modalities are generally associated with good outcomes and an improvement of HRQoL after SPKT*.* PD prior to SPKT seems to offer a slight advantage compared with HD before transplantation. Further prospective, controlled and randomized studies are needed to evaluate sociodemographic factors with an impact on the outcome and HRQoL.

## Data Availability

Our database contains highly sensible data which may provide insight in clinical and personnel information about our patients and lead to identification of these patients. Therefore, according to organizational restrictions and regulations these data cannot be made publically available. However, the datasets used and/or analyzed during the current study are available from the corresponding author on reasonable request.

## Abbreviations

BMI: Body mass index
ESRD: End stage renal disease
HD: Hemodialysis
HRQoL: Health-related quality of life
KTA: Kidney transplantation alone
MMF: Mycophenolate mofetil
PD: Peritoneal dialysis
RRT: Renal replacement therapy
SD: Standard deviation
SF-36: Short Form 36
SPKT: Simultaneous pancreas-kidney transplantation
